# The Essential Oil from the Roots of *Valeriana rigida* Ruiz & Pav. Growing in the Paramos of Chimborazo (Ecuador): Chemical Analysis, Enantioselective Profile, and Preliminary Biological Activity

**DOI:** 10.3390/plants14071062

**Published:** 2025-03-29

**Authors:** Linda M. Flores, Diego R. Vinueza, Gianluca Gilardoni, Antonio J. Mota, Omar Malagón

**Affiliations:** 1 Facultad de Ciencias, Escuela Superior Politécnica de Chimborazo (ESPOCH), Panamericana Sur Km 1 ½, Riobamba 060155, Ecuador; linda.flores@espoch.edu.ec (L.M.F.); drvinueza@espoch.edu.ec (D.R.V.); 2 Departamento de Química, Facultad de Ciencias, Campus Fuentenueva, Universidad de Granada, 18071 Granada, Spain; 3 Departamento de Química, Universidad Técnica Particular de Loja (UTPL), Calle Paris s/n y Praga, Loja 110107, Ecuador; ggilardoni@utpl.edu.ec; 4 Programa de Doctorado en Química, Universidad Técnica Particular de Loja (UTPL), Calle Paris s/n y Praga, Loja 110107, Ecuador; 5 Departamento de Química Inorgánica, Facultad de Ciencias, Campus Fuentenueva, Universidad de Granada, 18071 Granada, Spain; mota@ugr.es

**Keywords:** *Valeriana rigida*, essential oil, enantiomers, chiral separation, neutrophils, anti-inflammatory activity

## Abstract

The essential oil (EO) obtained from the roots of *Valeriana rigida* Ruiz & Pav. (Caprifoliaceae), collected in the moorland region of Chimborazo Province, Ecuador, was analyzed for the first time. The chemical profile was qualitatively and quantitatively analyzed using GC-MS and GC-FID, respectively. With both detectors, two stationary phases of different polarities were used. A total of 56 compounds were identified, and the most abundant components (>3% on at least one column) were a mixture of cyclosativene and α-ylangene (4.5–4.4%), α-copaene (9.0–8.8%), decanoic acid (16.0–15.6%), β-chamigrene (3.2–3.1%), δ-cadinene (9.7–9.5%), dodecanoic acid (13.4–12.3%), and 7-*epi*-α-eudesmol (5.0–4.9%), on a non-polar and polar stationary phase, respectively. Additionally, the enantioselective analysis showed (1*S*,5*S*)-(+)-α-pinene, (1*R*,4*S*)-(–)-camphene, (1*S*,5*S*)-(−)-β-pinene, and (1*R*,2*S*,6*S*,7*S*,8*S*)-(–)-α-copaene as enantiomerically pure compounds, whereas germacrene D exhibited both enantiomeric forms. The anti-inflammatory activity of *V. rigida* EO was comparable to that of aspirin, as indicated by the IC50 values, with no significant differences observed.

## 1. Introduction

Human beings have maintained a close relationship with plants for a long time, which has allowed them to acquire a rich collection of botanical knowledge. The use of plant-derived bioactive compounds has been essential for the development and application of pharmaceuticals throughout history. Most research on active molecules has been conducted in subtropical regions. However, tropical areas exhibit greater biological diversity, as is the case in Ecuador. This is why our studies focus on regions with high biological diversity, and the paramos, due to their harsh conditions, may be a valuable source of secondary metabolites of interest.

Ecuador is considered a megadiverse country [1], with a wide variety of plant species, due to a combination of geological, ecological, and evolutionary factors. It hosts approximately 10% of the world’s plant species [2], distributed across four regions, at altitudes ranging from the sea level to 6300 m. The vegetation varies from xerophilous shrubs to rainforests and high-altitude moorlands, which has sparked interest in the analysis of its secondary metabolites [3,4,5]. As of 2016, 263 molecules were reported, isolated from 58 families, with notable examples including Asteraceae, Solanaceae, Orchidaceae, and Lamiaceae, among others [6].

Andean moorlands or paramos are humid tropical ecosystems, found in the high-altitude regions of mountains, ranging from 3000 to 4700 m above sea level. Like the alpine tundra, it is characterized by herbaceous vegetation and shrubs [7]. They contain approximately 6.7% of the world’s endemic species [8]. Paramos are capable of supplying water and capturing carbon [9,10], due to the relationship between biodiversity and ecosystem functioning [11]. Ecuadorian paramos host approximately 628 endemic plant species, representing 4% of the country’s total flora [12]. They are located along the Andean corridor [13], covering an area of 1,833,834 hectares, which represents about 5% of the national territory [14]. It extends from the northern border with Colombia to the southern border with Peru, spanning about 600 km in length. Due to the harsh conditions in these areas, plants have developed survival mechanisms [15]. The Chimborazo province has an area of 649,970 hectares, of which 42% (273,660 hectares) belong to the high-altitude moorland ecosystem [16].

In 2009, the Angiosperm Phylogeny Group (APG III) classified flowering plants, placing the family *Valerianaceae* within *Caprifoliaceae* [17]. Years later, in 2016, the Angiosperm Phylogeny Group (APG IV) reaffirmed the classification of *Valerianaceae* as part of *Caprifoliaceae* [18]. A total of 960 species are currently classified within *Caprifoliaceae* [19], distributed worldwide, with the highest diversity found in East Asia and North America. [20].

Among the many Ecuadorian plants for which secondary metabolism is still unstudied, the highlander species *Valeriana rigida* Ruiz & Pav. must be mentioned. This taxon is also registered with the synonym *Phyllactis rigida* (Ruiz & Pav.) Pers [21], of which the genus includes the three species *P. rigida*, *P. pulvinata*, and *P. dorotheae*.[22]. The species *V. rigida*, commonly known as “Valeriana estrella”, is widely distributed in the high-altitude regions of Ecuador, ranging from 2500 to 4000 a.s.l., across the provinces of Azuay, Bolívar, Carchi, Chimborazo, Cotopaxi, Imbabura, Loja, Napo, and Pichincha [23]. The traditional uses of *V. rigida* include infusions to treat conditions, such as insomnia, relaxation, nervous disorders, headaches, and menopause [24,25]. These uses align with the sedative and anxiolytic properties reported in several species of the same genus [26,27,28]. The iridoid compounds responsible for the sedative effect found in *Valeriana* spp. are known as valepotriates, such as valtrate, isovaltrate, acevaltrate, and dihydrovaltrate [29]. Previous studies on the essential oils (EOs) from different species of the genus *Valeriana* have shown anti-inflammatory activity, like in the case *V. jatamansi* [30]. Aqueous leaf extracts of *V. wallichii* at a dose of 200 mg/kg have also demonstrated effectiveness in anti-inflammatory activity [31]. Essential oils are oily, volatile substances with a strong and specific odor, obtained through distillation processes using boiling water, steam, or mechanical processing. They exhibit various biological activities, including antibacterial, anti-inflammatory, antioxidant, among others [32].

The objective of the present study is to investigate the EO distilled from the roots of *V. rigida*. In addition to the chemical composition of the volatile fraction, this research was complemented with the study of its anti-inflammatory activity using activated neutrophils. Finally, an enantioselective analysis of some major chiral components was performed, to determine their enantiomeric excesses and, according to the literature, their stereoselective biological properties. To the best of the authors’ knowledge, this is the first chemical and enantioselective investigation of an essential oil from *V. rigida*.

## 2. Results

### 2.1. Chemical Composition of the EO

The essential oil was distilled from the roots of *V. rigida*, with an average yield of 0.03 (*w/w*), calculated from three repetitions.

**Figure 1:**
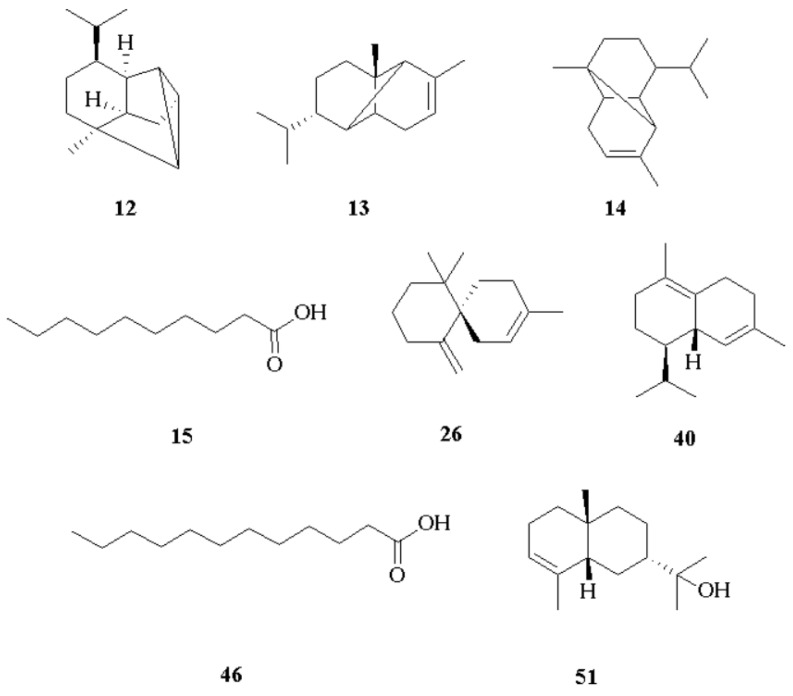
Main compounds identified in the root essential oil of *V. rigida:* cyclosativene, α-ylangene (**12**,**13**), α-copaene (**14**), decanoic acid (**15**), β-chamigrene (**26**), δ-cadinene (**40**), dodecanoic acid (**46**), and 7-*epi*-α-eudesmol (**51**).

A total of 56 compounds were identified via GC-MS and quantified via GC-FID. The major components of the volatile fraction (>3% on at least one column) were a mixture of the inseparable cyclosativene and α-ylangene (**12** and **13**) (4.5–4.4%), α-copaene (**14**) (9.0–8.8%), decanoic acid (**15**) (16.0–15.6%), β-chamigrene (**26**) (3.2–3.1%), δ-cadinene (**40**) (9.7–9.5%), dodecanoic acid (**46**) (13.4–12.3%), and 7-*epi*-α-eudesmol (**51**) (5.0–4.9%), as shown in Figure 1. All the identified constituents represented 84.0% and 80.5% of the total EO. A standard deviation of less than 5% was obtained for the percentages of each analyte in both columns. The complete results are reported in Table 1.

**Figure 2:**
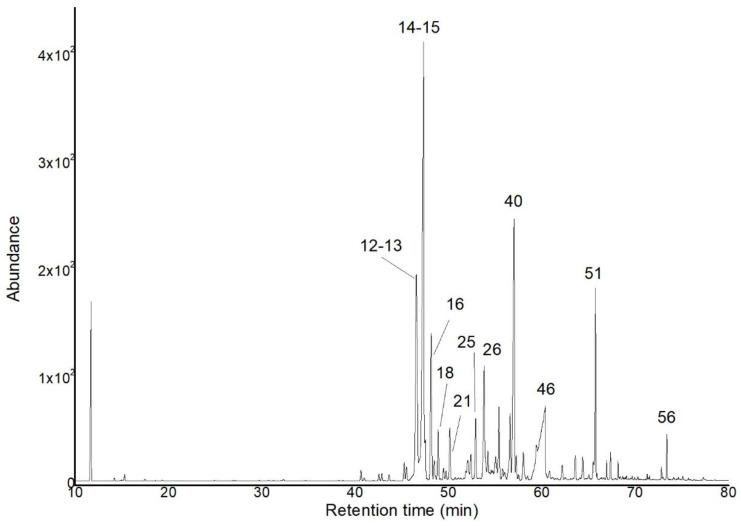
GC–MS chromatogram of the EO from the roots of *V. rigida* in a 5% phenyl-methylpolysiloxane-based column. The main components are presented in Table 1, according to the number corresponding to each peak.

**Figure 3:**
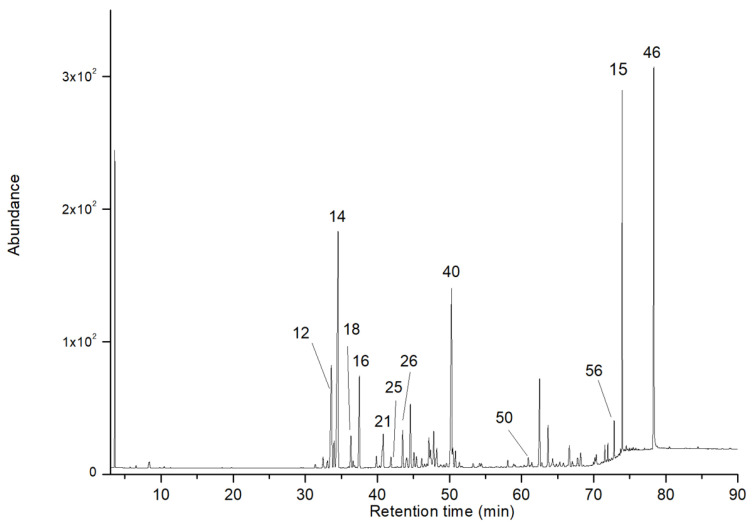
GC–MS chromatogram of the EO from the roots of *V. rigida* in a polyethylene glycol-based column. The main components are presented in Table 1, according to the number corresponding to each peak.

Sesquiterpene compounds constituted the highest percentage of the volatile fraction, accounting for 50.7–48.9% of the total weight, followed by 0.5–0.7% of monoterpene-type compounds. Finally, other components were present in 32.8–30.9%, highlighting the presence of acids, unsaturated aliphatic aldehydes, and unidentified compounds. The chromatographic profiles on the 5% phenyl methyl polysiloxane and polyethylene glycol stationary phases are shown in Figure 2 and Figure 3, respectively.

### 2.2. Enantioselective Analysis

The enantioselective analyses (Table 2) identified four enantiomerically pure compounds in the essential oil of *V. rigida*: (1S,5S)-(+)-α-pinene, (1R,4S)-(–)-camphene, (1S,5S)-(−)-β-pinene, and (1R,2S,6S,7S,8S)-(–)-α-copaene. In contrast, germacrene D presented both enantiomeric forms.

### 2.3. Anti-Inflammatory Activity

The inhibitory effect of *V. rigida* essential oil on isolated activated neutrophils (superoxide anion production) is summarized at Table 3, using water-soluble tetrazolium salt (WST-1) for detection.

The EO and aspirin (at doses ranging from 3.125 to 100 μg/mL) showed proportional anti-inflammatory effects on the tested model. The anti-inflammatory activity of the EO was comparable to that of aspirin at medium to high concentrations. Likewise, when the IC_50_ values were compared (38.17 ± 5.59 μg/mL for EO vs. 46.53 ± 3.50 μg/mL for aspirin), no significant differences were observed.

In addition to the anti-inflammatory activity assay, the EO was submitted to a radical scavenging, test based on the use of DPPH. Furthermore, no anti-radical activity was detected, and the DPPH assay will not be discussed further.

## 3. Discussion

### 3.1. Chemical Composition and Main Components

The average EO yield from the roots of *V. rigida* was 0.03 (*w/w*). It is important to note that the EO yield depends on various factors, including the plant species and the specific part used (buds, flowers, leaves, stems, twigs, seeds, fruits, roots, wood, or bark). According to the literature, the EO yield from the roots of *V. officinalis* ranges between 0.28% and 1.16%. Some studies have shown that the chemical profile of the volatile fraction obtained from *V. officinalis* roots can vary due to different geographical conditions in which the species developed, as well as the season in which it was collected [64,65].

For the first time, the chemical profile of the EO obtained from the roots of *V. rigida* was reported, with a total of 56 compounds identified. The chemical composition was compared with that of the EO from *V. officinalis* and *V. jatamansi*, as they are the most representative species of this genus.

Eight common compounds were identified in the EOs obtained from the roots of *V. rigida* and *V. officinalis*. Among them, δ-cadinene and cyclosativene exhibited a higher percentage in *V. rigida* (9.7–9.5% and 4.5–4.4% respectively) compared to *V. officinalis* (1.51–1.87% and 0.84–0.38%). Other compounds, such as α-humulene and (*E*)-β-caryophyllene, were present in both species at similar percentages. The former was quantified as 0.4% in *V. rigida* and 0.58–8.46% in *V. officinalis*, while the latter was present at 1.1–1.3% in *V. rigida* and (1.46–1.87%) in *V. officinalis* [66]. Finally, some compounds were found in lower percentages in *V. rigida*. Camphene and α-pinene were respectively present at 0.1% compared to 4.96–7.19% and 1.82–3.83% in *V. officinalis*. The chemical composition of essential oils varies between species and is influenced by factors, such as the geographical location [67], environmental conditions [68,69,70], maturity stage [71], and extraction method [71,72].

Four common compounds were identified in the EOs of *V. rigida* and *V. jatamansi*: α-pinene, camphene, α-humulene, and viridiflorol. The percentage of α-pinene in *V. rigida* was 0.1%, while in *V. jatamansi*, it reached an average amount of 0.59%. Camphene was present at 0.1–0.2% in *V. rigida*, being higher in *V. jatamansi* (0.77%). α-Humulene was found at 0.4% in *V. rigida*, significantly lower compared to the value of 2.25% in *V. jatamansi*. Finally, viridiflorol was slightly higher in *V. jatamansi*, with 0.605% compared to (0.4–0.2% in *V. rigida* [73]. These results highlighted a chemical relationship between both species, reflecting similar patterns despite their ecological and taxonomic differences.

It is evident that the chemical profile of these species contained monoterpenes, sesquiterpenes, and other compounds, with sesquiterpenes predominating and monoterpenes present in smaller percentages. It is common to find aliphatic carboxylic acids in the chemical profile of *Valeriana* spp. EOs [74,75,76]. Valeric acid is one of the most characteristic components; with the molecular formula C_5_H_10_O_2_ and a linear structure, it is one of the key acids, possibly contributing to the relaxing effect of these plants [77]. In *V. officinalis*, it can reach 0.2% of the whole oil mass [78]. Another characteristic acid is isovaleric acid, which is generated as a product of the hydrolysis of valepotriates [79]. The EO obtained from the roots of *V. officinalis* contains up to 44.6% of isovaleric acid [78], while the volatile fraction of *V. pilosa* has a higher percentage (2.6%) [78]. Finally, long-chain carboxylic acids are not so common in all *Valeriana* species; for instance, in *V. officinalis* they can reach 11.4%, whereas *V. hardwickii* EO does not contain these organic acids [80].

Another species where long-chain carboxylic acids were described is *V. alliariifolia*, a species from the Venezuelan paramos, in which decanoic acid (0.1%), dodecanoic acid (0.5%), and hexadecanoic acid (4.3%) have been detected [81]. On the other hand, in *V. rigida* EO, decanoic (16.0%) and dodecanoic (13.4%) acids were present in significantly higher percentages, whereas hexadecanoic acid did not exceed 0.5%. No species within the *Valerianaceae* and *Caprifoliaceae* families have been reported to contain decanoic and dodecanoic acids above 0.5%. Therefore, *V. rigida* is the first species to be documented with such high levels of these acids. Finally, there are species for which the chemical profile lacks the presence of organic acids [73,82,83].

### 3.2. Enantioselective GC-MS Analysis of Enantiomeric Distribution

The enantioselective GC-MS analysis was performed to determine the enantiomeric composition of five chiral compounds, present in the essential oil of *V. rigida*. The determination of the enantiomeric composition is a powerful tool for authenticating essential oils, as oils derived from different plants can be adulterated through the addition of foreign components [84,85].

Silva et al. evaluated the antimicrobial properties of α- and β-pinene enantiomers against *C. albicans*, *Cryptococcus neoformans*, *Rhizopus oryzae*, and methicillin-resistant *S. aureus* (MRSA). Their results revealed that the negative enantiomers showed no antimicrobial activity up to a concentration of 20 µg/mL, whereas the positive enantiomers exhibited a potent capacity, eliminating 100% of *C. albicans* within 60 min [86]. Dhar et al. also evaluated α-pinene enantiomers against Gram-positive bacteria (*Micrococcus luteus* and *S. aureus*), Gram-negative bacteria (*E. coli*), and a fungus (*C. albicans*), demonstrating that (+)-α-pinene exhibited a modest action against the selected microbes, while (−)-α-pinene showed no activity [87].

No information has been found in the literature regarding the enantiomeric properties of camphene, as its enantiomers are not abundant in nature. Biological research has demonstrated that camphene exhibits various biological activities both in vitro and in vivo, including antibacterial [88], antifungal [89], antioxidant [88], and anti-inflammatory effects [90]. It has also been reported that camphene possesses acetylcholinesterase inhibitory properties [91].

Despite copaene having no studies regarding the biological activity of its enantiomers, Alfonso et al. analyzed the influence of α-copaene on the susceptibility of *Olea europaea* L. towards the olive fly *Bactrocera oleae* (Rossi). In that study, the authors demonstrated that fruits with a higher amount of (+)-α-copaene favored the oviposition of *B. oleae* females, whereas an increase in (−)-α-copaene did not produce the same effect [92].

Finally, the effects of germacrene D on insect behavior are known [93]. It is also known that the enantiomers (+)- and (–)-germacrene D have different bioactivities. The germacrene D receptor neurons in heliothine moths exhibited a 10-fold higher affinity for (−)-germacrene D compared to (+)-germacrene D. This is due to the greater sensitivity towards (−)-germacrene D, as this enantiomer predominates in higher plants [94].

### 3.3. Anti-Inflammatory Activity

The anti-inflammatory activity of *V. rigida* essential oil was comparable to the gold-standard aspirin under the tested model conditions. This finding is supported by results from a series of previously published studies [95,96,97,98], which have reported the neuroprotective effects of various chemical compounds from related species within the *Valeriana* genus. Given that neuroinflammation plays a key role in the progression of neurodegenerative diseases, it is plausible that the observed neuroprotection is, at least in part, mediated by the anti-inflammatory potential of these compounds [29]. In the present study, aspirin was used as a positive control since, according to literature, non-steroidal anti-inflammatory drugs (NSAIDs) inhibit neutrophil functions, through mechanisms independent of their effects on prostaglandin biosynthesis [98].

Although the anti-inflammatory activity of *Valeriana* species has been well-documented, this study represents the first report recognizing the anti-inflammatory properties of *V. rigida* essential oil. Among the key compounds identified, cyclosativene has been proposed as a natural product with antioxidant properties that may help mitigate oxidative damage in the context of neurodegenerative disorders [99].

While the activity of α-ylangene has not been extensively reported as an isolated compound, studies have shown that essential oils enriched in this chemical possess antioxidant, anti-inflammatory, anticancer, and antimicrobial activities [100]. Similarly, α-copaene and δ-cadinene are common compounds found in the essential oils of various plants known for their anti-inflammatory, antioxidant, anticancer [101,102,103], and neuroprotective properties [104,105,106].

Studies on β-chamigrene, often derived from the synthesis of this compound, have shown its correlation with antioxidant, anti-inflammatory, and analgesic effects when found in extracts from Mango stem bark [107]. Furthermore, its antioxidant and antibacterial properties have been documented [108], as well as its antimicrobial and cytotoxic effects on cancer cell lines [109]. Additionally, 7-epi-α-eudesmol, a constituent of essential oils, has demonstrated antioxidant, anti-inflammatory, and cytotoxic properties [110], as well as anti-acetylcholinesterase activity [111].

While several isolated compounds from *V. rigida* essential oil have shown anti-inflammatory effects, which have been reported to reduce the production of nitric oxide (NO), interleukin-6 (IL-6), and tumor necrosis factor-α (TNF-α) [112], the observed biological activity could more precisely be attributed to a phytocomplex with synergistic effects [113].

## 4. Materials and Methods

### 4.1. Plant Material

The roots of *V. rigida* were collected on January 15, 2024, within a 200 m range around a reference point with coordinates of 1°29′2.79″ S and 78°45′34.95″ W, at an altitude of 4200 m above sea level. After collection, the roots were dried at 35 °C for 48 h and stored in a dark, cool place until further use. After identification, a botanical specimen was deposited at the herbarium of the Escuela Superior Politécnica de Chimborazo (ESPOCH) under the reference code No. 0.15. CHEP. 2024. This research was conducted with permission from the Ministry of Environment, Water, and Ecological Transition of Ecuador, under registration number MAATE-ARSFC-2024-0161.

### 4.2. Sample Preparation and EO Distillation

An amount of 2.1 kg of dried root was ground and divided into three samples of 700 g each, which were subjected to a 5 h steam distillation in a modified Dean–Stark apparatus. At the end of the process, 0.188 g, 0.211 g, and 0.193 g of essential oil were obtained. The oils were dehydrated with anhydrous sodium sulfate, purchased from Merck (Sigma–Aldrich, St. Louis, MO, USA), and stored at −15 °C for further use. Analytical samples for GC analysis were prepared following the methods described in the literature, using *n*-nonane as an internal standard [114,115,116,117,118].

### 4.3. Qualitative Chemical Analysis

The qualitative analysis was carried out using a gas chromatography (GC) instrument model Trace 1310, equipped with a single quadrupole ISQ 7000 mass spectrometry (MS) detector (Thermo Fisher Scientific, Waltham, MA, USA). In this GC, 1 µL of the analytical sample was injected in split mode (split ratio 40:1). The injector temperature was set at 250 °C, with helium (provided by Indura S.A., Guayaquil, Ecuador) as the carrier gas, programmed at the constant flow of 1 mL/min. The ionization was conducted based on electron impact, at an ionization energy of 70 eV. The mass analyzer was operated in SCAN mode, with a mass range of 40–400 *m/z*. This analysis was repeated on two columns with stationary phases of different polarities: the non-polar 5%-phenyl methyl polysiloxane (TR-5ms) and the polar polyethylene glycol (TR-WAX), both purchased from Thermo Fisher Scientific (Waltham, MA, USA). The two columns were 30 m long, with an internal diameter of 0.25 mm and a film thickness of 0.25 μm. The TR-5ms column operated under the following thermal conditions: it started at 60 °C for 5 min, followed by a first thermal gradient reaching 100 °C at a rate of 2 °C/min, a second gradient reaching 150 °C at 3 °C/min, and a third gradient reaching 200 °C at 5 °C/min. Finally, the oven was maintained at a temperature of 250 °C for 15 min after a thermal gradient of 15 °C/min. For the TR-WAX column, the same thermal conditions and oven program were applied, except for the final temperature, which was set at 230 °C. For the identification of the components present in the EO of *V. rigida*, each mass spectrum was compared, along with its linear retention index (LRI), with data from literature. A series of *n*-alkanes from C_9_ to C_25_ (Sigma–Aldrich, St. Louis, MO, USA) was used to calculate the LRI of each component, according to Van Den Dool and Kratz [119].

### 4.4. GC-FID Quantitative Analyses

The quantitative analysis was performed using the same GC instrument as the qualitative one, but with a flame ionization detector (GC-FID). The thermal conditions, columns, gas flow, and injection parameters were the same as in the qualitative analysis. For quantification, the relative response factor (RRF) of each component was calculated against isopropyl caproate, based on the combustion enthalpy [120,121]. A six-point calibration curve was constructed for each column, using isopropyl caproate (synthesized and purified by the authors with a GC purity of 98.8%) as the calibration standard and *n*-nonane (Sigma–Aldrich, St. Louis, MO, USA) as the internal standard, according to the literature [122]. Both curves yielded a correlation coefficient > 0.999.

### 4.5. Enantioselective Analysis of the EO

For the enantioselective analysis, GC-MS was also used with two enantioselective columns, of which the stationary phases were based on 2,3-diacetyl-6-*tert*-butyldimethylsilyl-β-cyclodextrin and 2,3-diethyl-6-*tert*-butyldimethylsilyl-β-cyclodextrin (Mega, Milan, Italy). Both columns were 25 m in length, 250 μm in internal diameter, and 0.25 μm in phase thickness. The injector temperature, carrier gas flow, and MS parameters were the same as the ones used in the qualitative analysis, while the injector was operated in split mode (split ratio 50:1). The following thermal program was applied: an initial temperature of 60 °C for 2 min, followed by a temperature gradient of 2 °C/min up to 220 °C, which was maintained for 2 min. To determine the LRIs, a mixture of *n*-alkanes from C_9_ to C_25_ (Sigma–Aldrich, St. Louis, MO, USA) was also injected, according to Van den Dool and Kratz. The enantiomers were identified based on their mass spectra and elution order, after injecting enantiomerically pure standards.

### 4.6. Oxidative-Burst Assay (Antiinflammatory)

To screen the anti-inflammatory activity of *V. rigida* essential oil, the oxidative burst assay developed by Tan and Berridge [110] was utilized, with slight modifications by Vinueza et al. [111]. Briefly, after a heparinized fresh venous blood sample was drawn from a healthy volunteer, the whole blood was diluted at a ratio (1:1) with Hanks’ Balanced Salt Solution (HBSS). Later, ficoll paque™ PLUS was added at a ratio (3:4) regarding the diluted blood sample; then, it was centrifuged for 30 min at 1500 rpm. After discarding the supernatant, red blood cell traces were lysed by mixing with a hypotonic ammonium chloride solution (0.83% *w/v*). Finally, the sample was centrifuged again, and the neutrophils were washed with HBSS pH 7.4 and resuspended at a concentration of 10^7^ cells/mL in an appropriate volume of HBSS.

Anti-inflammatory activity was determined as a function of the reduction of water-soluble tetrazolium salt (WST-1) in the presence of activated neutrophils. The assay was carried out in a total volume of 250 µL of HBSS (pH 7.4) containing 10^7^ neutrophils/mL, 500 µM WST-1, and various concentrations of essential oil (3.125, 6.25, 12.5, 25, 50, and 100 µg/mL) or aspirin, which was used as the reference compound, both dissolved with HBSS enrichment with DMSO, such that the final concentration of DMSO in each well was 0.05%. The control contained HBSS, a neutrophil suspension, and WST-1. All compounds were equilibrated at 37 °C and the reaction was initiated by adding opsonized Zymosan A (15 mg/mL), which was prepared by mixing it with human pooled serum, followed by centrifugation at 3000 rpm, and the pellet was suspended in HBSS. Absorbance was measured at 450 nm. DMSO (0.05% *v/v*) together with the neutrophil suspension and opsonized Zymosan A aliquot were used as a blank, and the anti-inflammatory activity was expressed as the produced superoxide anion inhibition percent.

## Figures and Tables

**Table 1:** Chemical analysis of *V. rigida* root essential oil on two stationary phases of different polarities.

	Compounds	5% Phenyl Methyl Polysiloxane				Polyethylene Glycol				Reference
		LRI ^a^	LRI ^b^	%	σ	LRI ^a^	LRI ^c^	%	σ	
1	α-pinene	929	932	0.1	0.03	1019	1026	Trace	-	[33]
2	camphene	943	946	0.1	0.07	1060	1060	0.2	0.05	[34]
3	valeric acid	946	939	0.1	0.06	1693	-	0.2	0.08	§
4	β-pinene	970	974	Trace	-	1106	1105	0.0	0.01	[35]
5	2-methoxy-3-(1-methylpropyl)-pyrazine	1168	1168	0.1	0.05	1505	1509	0.1	0.01	[36]
6	isobornyl acetate	1280	1283	0.3	0.12	1582	1577	0.5	0.10	[37]
7	(*2E*,*4Z*)-decadienal	1287	1292	0.1	0.01	1776	1779	0.2	0.02	[38]
8	(*E*,*E*)-2,4-decadienal	1309	1315	0.2	0.14	1810	1814	0.2	0.01	[39]
9	unidentified (MW = 196)	1315	-	0.2	0.09	1587	-	0.5	0.12	§
10	8,9-didehydrocycloisolongifolene	1321	1317	0.1	0.07	1436	-	0.1	0.02	§
11	α-cubebene	1345	1348	0.5	0.09	1450	1450	0.3	0.03	[40]
12	cyclosativene	1360	1369	4.5	0.80	1466	1465	4.4	0.70	[41]
13	α-ylangene	1362	1373			1468	1470			[42]
14	α-copaene	1373	1374	9.0	2.44	1481	1483	8.8	2.20	[43]
15	decanoic acid	1378	1382	16.0	0.02	2297	2294	15.6	3.57	[44]
16	β-cubebene	1386	1387	2.9	0.19	1530	1527	2.8	0.16	[42]
17	7-*epi*-sesquithujene	1390	1390	0.4	0.09	1570	-	0.3	0.06	§
18	cyperene	1394	1398	1.1	0.42	1510	1514	1.1	0.25	[45]
19	β-longipinene	1404	1400	0.4	0.13	1516	-	0.2	0.05	§
20	β-funebrene	1410	1413	0.2	0.07	1532	1618	Trace	-	[46]
21	(*E*)-β-caryophyllene	1413	1420	1.1	0.52	1583	1585	1.3	0.29	[47]
22	β-gurjunene	1423	1431	0.1	0.03	1578	1580	0.1	0.01	[48]
23	α-guayen	1444	1437	0.3	0.12	1604	1604	0.3	0.06	[49]
24	α-humulene	1447	1452	0.4	0.06	1656	1656	0.4	0.09	[50]
25	alloaromadendrene	1455	1458	1.1	0.25	1631	1631	0.9	0.20	[51]
26	β-chamigrene	1469	1476	3.2	1.01	1650	-	3.1	0.97	§
27	γ-muurolene	1472	1478	0.1	0.06	1699	1702	0.5	0.13	[52]
28	germacrene D	1475	1480	0.8	0.30	1679	1678	0.9	0.16	[53]
29	widdra-2,4(14)-diene	1478	1481	0.2	0.05	1568	-	0.3	0.07	§
30	unidentified (MW = 204)	1480	-	0.2	0.07	1705	-	0.1	0.07	-
31	unidentified (MW = 204)	1485	-	0.2	0.14	-	-	Trace	-	-
32	*cis*-β-guaiene	1487	1492	0.3	0.03	1678	1671	0.3	0.05	[54]
33	epicubebol	1489	1493	0.4	0.05	1938	1928	0.5	0.09	[55]
34	eciphyllene	1494	1501	2.1	0.80	1705	-	1.1	0.31	§
35	*trans*-β-guaiene	1495	1639			1474	-	0.8	0.36	§
36	premnaspirodiene	1499	1505	0.4	0.12	1664	-	0.3	0.07	§
37	unidentified (MW = 204)	1502	-	0.1	0.04	1794	-	0.2	0.02	-
38	cubebol	1510	1514	0.3	0.14	1939	1930	0.8	0.13	[56]
39	unidentified (MW = 220)	1512	-	1.7	0.35	2000	-	1.3	0.11	-
40	δ-cadinene	1520	1522	9.7	0.70	1750	1752	9.5	1.25	[57]
41	(*E*)-iso-γ-bisabolene	1522	1529	0.7	0.20	1760	1762	0.4	0.08	[58]
42	γ-cuprenene	1527	1532	0.2	0.02	2060	-	0.2	0.02	§
43	α-copaen-11-ol	1535	1539	0.9	0.12	2053	-	0.7	0.11	§
44	silphiperfol-5-en-3-one B	1541	1550	0.2	0.03	1944	-	0.3	0.07	§
45	β-calacorene	1557	1564	0.1	0.04	1907	1912	0.1	0.04	[59]
46	dodecanoic acid	1569	1567	13.4	0.95	2485	2487	12.3	1.33	[60]
47	viridiflorol	1597	1592	0.4	0.03	2183	-	0.2	0.02	§
48	silphiperfol-6-en-5-one	1622	1624	0.7	0.13	2080	-	0.5	0.03	§
49	alloaromadendrene oxide	1636	1639	0.8	0.20	2065	-	0.3	0.02	§
50	unidentified (MW = 202)	1666	-	0.2	0.22	2008	-	0.4	0.04	-
51	7-*epi*-α-eudesmol	1669	1662	5.0	0.87	1740	-	4.9	0.77	§
52	pentadecanal	1710	1716	0.5	0.07	2029	2024	0.2	0.04	[61]
53	unidentified (MW = 200)	1723	-	0.4	0.28	2201	-	0.4	0.04	-
54	unidentified (MW = 204)	1746	-	0.1	0.00	2141	-	0.4	0.02	-
55	hexadecanoic acid	1959	1959	0.5	0.18	2923	2928	0.3	0.12	[62]
56	3-(*Z*)-cembrene A	1966	1965	0.9	0.11	2242	-	0.7	0.11	§
	monoterpene hydrocarbons			0.2				0.2		
	oxygenated monoterpenes			0.3				0.5		
	sesquiterpene hydrocarbons			41.5				40.5		
	oxygenated sesquiterpenes			8.3				7.7		
	diterpene sesquiterpenes			0.9				0.7		
	others			32.8				30.9		
	total			84.0				80.5		

^a^ Calculated linear retention index; ^b^ linear retention index according to [63]; ^c^ linear retention index based on reference (Ref.); trace < 0.1%; MW = molecular weight; § = identification based on MS only.

**Table 2:** Enantiomeric separations with two cyclodextrin-based enantioselective columns.

Chiral Selector	Enantiomer	LRI	Enantiomeric Distribution %	e.e. %
DAC	(1*S*,5*S*)-(−)-α-pinene	926	-	100
DAC	(1*S*,5*S*)-(+)-α-pinene	929	100	
DET	(1*R*,4*S*)-(+)-camphene	932	-	100
DET	(1*R*,4*S*)-(–)-camphene	922	100	
DET	(1*S*,5*S*)-(−)-β-pinene	961	100	100
DET	(1*S*,5*S*)-(+)-β-pinene	944	-	
DET	(1*R*,2*S*,6*S*,7*S*,8*S*)-(–)-α-copaene	1323	100	100
DET	(1*R*,2*S*,6*S*,7*S*,8*S*)-(+)-α-copaene	1319	-	
DET	(*R*)-(+)-germacrene D	1461	22.58	54.83
DET	(*S*)-(−)-germacrene D	1467	77.42	

DAC = 2,3-diacetyl-6-*tert*-butyldimethylsilyl-β-cyclodextrin; DET = 2,3-diethyl-6-*tert*-butyldimethylsilyl-β-cyclodextrin; LRI = linear retention index; e.e. = enantiomeric excess.

**Table 3:** Anti-inflammatory effect of *V. rigida* essential oil based on an isolated neutrophil model using water-soluble tetrazolium salt (WST-1).

Concentration (µg/mL)	*V. rigida* Essential Oil	Aspirin
3.1	2.32 ± 0.28	15.12 ± 2.07 **
6.2	6.26 ± 0.97	23.81 ± 3.21 **
12.5	24.46 ± 1.91	31.16 ± 2.97 *
25.0	46.39 ± 1.40	44.75 ± 4.74
50.0	51.42 ± 1.07	55.28 ± 2.30
100.0	61.42 ± 2.13	68.52 ± 5.28

The values represent the mean ± SD, n = 3. Significant values, ** *p* < 0.01, * *p* < 0.05, using Student’s *t*-test; *V. rigida* essential oil versus aspirin.

## Data Availability

The datasets presented in this article are not readily available because they are part of an ongoing study. Requests to access the datasets should be directed to the corresponding author.
